# High-quality single amplicon sequencing method for illumina MiSeq platform using pool of ‘N’ (0–10) spacer-linked target specific primers without PhiX spike-in

**DOI:** 10.1186/s12864-023-09233-4

**Published:** 2023-03-23

**Authors:** Tejali Naik, Mohak Sharda, Lakshminarayanan C P, Kumar Virbhadra, Awadhesh Pandit

**Affiliations:** 1 National Centre for Biological Science, Tata Institute of Fundamental Research, Bengaluru, Karnataka 560065 India; 2 School of Life Science, The University of Trans-Disciplinary Health Sciences & Technology (TDU), Bengaluru, Karnataka 560064 India

**Keywords:** Amplicon sequencing, ‘N’ (0–10) spacer-linked primer pool, Illumina, PhiX spike-in

## Abstract

**Background:**

Illumina sequencing platform requires base diversity in the initial 11 cycles for efficient cluster identification and colour matrix estimation. This limitation yields low-quality data for amplicon libraries having homogeneous base composition. Spike-in of PhiX library ensures base diversity but reduces the overall number of sequencing reads for data analysis. To overcome such low diversity issues during amplicon sequencing on illumina platforms, we developed a high throughput single amplicon sequencing method by introducing ‘N’ (0–10) spacers in target gene amplification primers that are pooled for simple handling.

**Result:**

We evaluated the efficiency of ‘N’ (0–10) spacer-linked primers by targeting bacterial 16S V3-V4 region, demonstrating heterogeneous base library construction. The addition of ‘N’ (0–10) spacers causes sequencing frameshift at every base that leads to base diversity and produces heterogeneous high quality reads within a single amplicon library. We have written a python based command-line software,“MetReTrim”, to trim the ‘N’ (0–10) spacers from the raw reads (https://github.com/Mohak91/MetReTrim). We further demonstrated the accuracy of this method by comparative mock community analysis with standard illumina V3-V4 primer method. The ZymoBIOMICS™ microbial community DNA standard was used as a control for this study. We performed data analysisusing the DADA2 pipeline where taxonomy was assigned using SILVA database as reference. We observed no difference between the communities represented by our method and standard illumina V3-V4 primer method.

**Conclusion:**

This method eliminates the need for PhiX spike-in for single amplicon sequencing on illumina MiSeq platform. This allows for sequencing of more number of samples in a run and a reduction in the overall cost. Given that Illumina sequencing works on SBS chemistry irrespective of the platform (such as HiSeq, MiSeq, NextSeq, NovaSeq, etc.) we propose that this strategy of using ‘N’ (0–10) spacer-linked primer design can be adopted for generating high-quality single locus amplicon sequencing in a high throughput manner across the illumina platform subject to further validation.

**Supplementary Information:**

The online version contains supplementary material available at 10.1186/s12864-023-09233-4.

## Background

Amplicon sequencing is an important and widely used tool for inferring the presence of taxonomic groups in microbial communities, detecting genetic variation embedded in complex genetic backgrounds, and is far more cost-effective than non-targeted sequencing when large amounts of undesired genetic material are present [1, 2]. Illumina HiSeq and MiSeq sequencing platforms are extensively used for performing paired-end sequencing to generate millions of reads for amplified fragments of the 16S rRNA gene, the internal transcribed spacer (ITS) region and different marker genes [3]. Illumina’s sequencing-by-synthesis technology uses fluorescently labelled reversible terminator-bound dNTPs. The red laser illuminates A and C and the green laser illuminates G and T fluorophores. Different optical filters are employed to image and identify the four different nucleotides. The similar emission spectra of the fluorophores (A and C as well as G and T) and the resulting limitations of the filters to properly distinguish the bases increases the chances of low base call quality and rate of miscalls in sequences [4–6]. For effective template generation and accurate base-calling on illumina platforms, it is therefore required to have nucleotide diversity (equal proportions of A, C, G and T nucleotides) at each base position in a sequencing library [7, 8]. Libraries of low sequence diversity like 16S rRNA gene are highly homogenous and commonly spiked with a high-diversity library such as PhiX, to alleviate the problem of homogenous signals generated across the entire flow cell. However, it reduces the overall sequence read throughput and multiplexing options because of it being a non-target (PhiX) library [3, 7]. The base diversity in first few cycles, particularly in the first 11 bases of the amplicon, are crucial for the identification of the sequencing clusters on the flow cell and colour matrix estimation [3, 9] Even though the research field has progressed in successful sequencing of 16S rRNA with illumina V3–V4 region primers, the problems of a drop in the read quality and inherent error rate still remain unresolved [9]. Another approach to deal with this issue is by sequencing libraries tagged with heterogeneity spacers at the 5’ end of the target gene amplicon during library preparation. The heterogeneity spacers are short sequences linked to index adaptors or to the gene-specific amplification primers in the form of 0–7 bases. These spacers minimize the need for PhiX spike-in to 10% by introducing base complexity at the start of sequencing reads yielding high-quality sequencing and increased multiplexing capacity [3, 7, 10–12]. However, designing primers or index adaptors consisting varying length of heterogeneity spacer with unique sequences for different types of amplicon libraries is a complex process due to the fact that every base sequenced at a given time should contribute to diversity (A ~ 25%, T ~ 25%, G ~ 25%, C ~ 25%) during the sequencing run and also requires PhiX spike-in. The PhiX spike-in hinders the use of MiSeq and HiSeq platforms to great levels. Another drawback is handling more heterogeneity primer pairs instead of a single gene-specific primer pair. The strategy involving amplification of target gene with various combinations of primer pairs like 0–7, 1–6, 2-5 makes the experimental setup tedious and requires a minimum of 8 reactions per sample to be pooled for confirming base complexity [9].

**Fig. 1:**
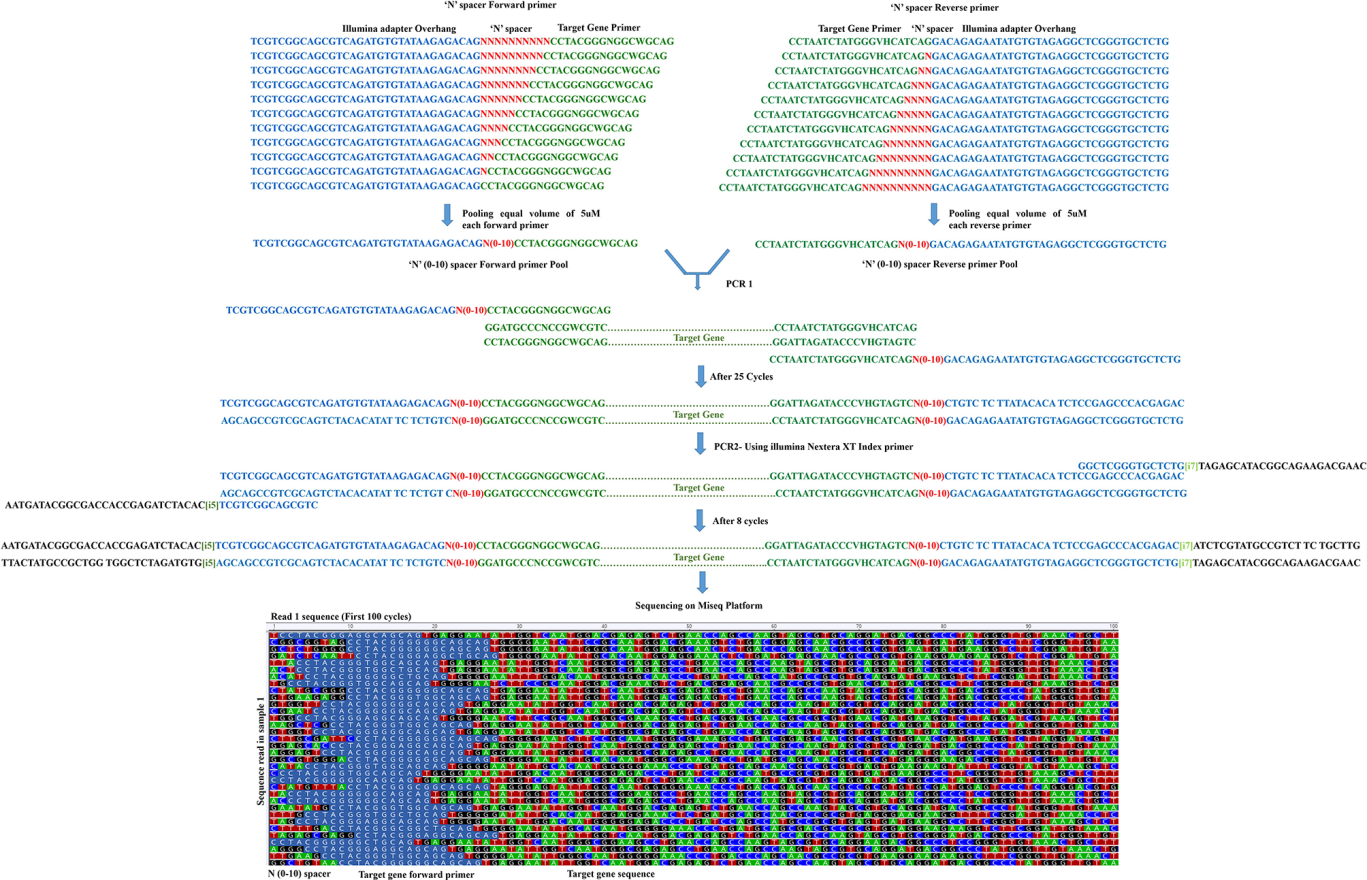
Schematic representation of 16 S amplicon sequencing workflow. *E. coli*/mock DNA was amplified for 16 S V3-V4 regions using pool of ‘N’ spacer-linked primers with overhang illumina adapters. The amplicons were subjected to second PCR using Nextera XT V2 index primers. Illumina MiSeq platform was used for sequencing the final libraries. The first 100 sequencing cycles illustrates the base diversity generated within a sample amplified using ‘N’ spacer-linked primers

To resolve the technical limitations of single amplicon sequencing on illumina platforms and challenges encountered during heterogeneity spacer primer designing, we added ‘N’ nucleotides to the 5’ end of the gene-specific primers for amplifying the gene. The ‘N’ nucleotide bases are added in 0–10 fashion in forward and reverse gene-specific primers. A pool of ‘N’ (0–10) spacers-linked gene-specific primers (Fig-1) is used for amplification and library synthesis incorporating diversity within a single library. In addition, the pool design reduces the number of primer combinations to a single set compared to previous studies [9, 13]. This strategy contributes to increased base diversity at each sequencing cycle in all the libraries that are multiplexed during a sequencing run on the Illumina platform.

To demonstrate the precision of our method we performed a comparative study with standard illumina V3-V4 primer method using a commercial mock community DNA standard (ZymoBIOMICS™). Towards this, we performed two independent runs and the generated reads were further analysed using DADA2 pipeline [14]. It has also been shown that the choice of reference database impacts the downstream analysis significantly [15]. In this study we used SILVA database as reference for taxonomic assignment. With the relative abundance data and correct taxa assignment, we demonstrated no significant differences between our method and standard illumina V3-V4 primer method. With this, we are introducing a universally adaptable method to perform single amplicon sequencing in illumina platforms without the need of PhiX spike-in.

## Results

The pilot experiments were designed to prepare libraries using ‘N’ spacer-linked primers from metagenomic DNA and *E. coli* DNA (Fig-1). The libraries were prepared from 5 sets of ‘N’ spacer-linked primer pool combinations (Table 1). The ‘N’ spacer-linked primer pool combinations were made by equimolar pooling of forward and reverse primer. Equimolar pool of barcoded libraries prepared using these primer pool combinations were denatured and spiked in Hiseq 2500 Rapid-V2 100 bp PE run to check base distribution at each sequencing cycle. Extracting fastq files from raw data, data de-multiplexing, and illumina adapter trimming was done using Bcl2fastq conversion software.

**Table 1:** ‘N’ spacer-linked primer pool combination

‘N’ spacer primer pool combinations	Forward Primer Pool	Reverse Primer Pool
6 N Spacer primer pool	6 N, 5 N, 4 N, 3 N, 2 N, 1 N, 0 N	6 N, 5 N, 4 N, 3 N, 2 N, 1 N, 0 N
7 N Spacer primer pool	7 N, 6 N, 5 N, 4 N, 3 N, 2 N, 1 N, 0 N	7 N, 6 N, 5 N, 4 N, 3 N, 2 N, 1 N, 0 N
8 N Spacer primer pool	8 N, 7 N, 6 N, 5 N, 4 N, 3 N, 2 N, 1 N, 0 N	8 N, 7 N, 6 N, 5 N, 4 N, 3 N, 2 N, 1 N, 0 N
9 N Spacer primer pool	9 N, 8 N, 7 N, 6 N, 5 N, 4 N, 3 N, 2 N, 1 N, 0 N	9 N, 8 N, 7 N, 6 N, 5 N, 4 N, 3 N, 2 N, 1 N, 0 N
10 N Spacer primer pool	10 N, 8 N, 7 N, 6 N, 5 N, 4 N, 3 N, 2 N, 1 N, 0 N	10 N, 8 N, 7 N, 6 N, 5 N, 4 N, 3 N, 2 N, 1 N, 0 N

The fastq files generated for metagenomic DNA and *E. coli* DNA were analyzed using an in-house python script to check for the diversity at each base position in read 1 and read 2 sequences. The read 1 sequence with ‘N’ (0–6) spacer and ‘N’ (0–7) spacer-linked primers (Fig-S1-A,B; Fig-S2-A,B) exhibited base diversity in the first ten nucleotides, allowing for better identification of clusters in the first few cycles, however at 11th and 12th base position, the contribution of “G”nucleotide is significantly high. Also, the base diversity pattern is similar for metagenomic and *E. coli* DNA. This confirms that the ‘N’ spacer-linked primer pool is able to generate base diversity in amplicon libraries from pure culture as well. Analysis of Read1 sequence for ‘N’ (0–8) and ‘N’ (0–9) spacer-linked primer pool (Fig-S1-C,D; Fig-S2-C,D) comparatively showed more promising base diversity but nucleotide distribution at position 15th -16th showed a bias towards green laser registry. Distribution of nucleotides for the green and red laser registry plays a critical role in obtaining good quality reads, therefore fastq results were analyzed for the ‘N’ (0–10) spacer-linked primer pool (Fig-S1-E; Fig-S2-E). We found that the ‘N’ (0–10) spacer-linked primer pool combination, although showed a significant increase in G nucleotide beyond the 12th base position, was balanced by an elevated percentage of A and C nucleotides responsible for the Red laser registry.

**Fig. 2:**
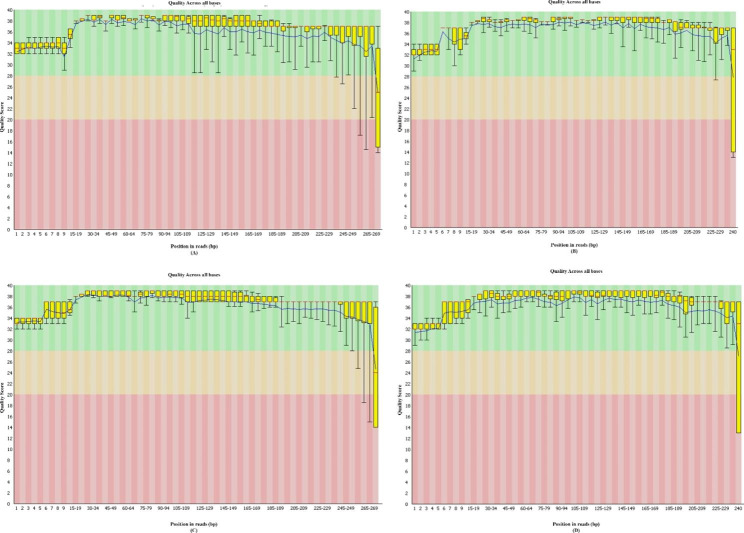
(A) - Per base sequence quality (read 1) of libraries prepared using Standard illumina V3-V4 primers with 20% PhiX spike-in. (B) - Per base sequence quality (read 2) of libraries prepared using Standard illumina V3-V4 primers with 20% PhiX spike-in. (C) – Per base sequence quality (read 1) of libraries prepared using ‘N’ (0–10) spacer-linked V3-V4 primers without PhiX Spike-in. (D) - Per base sequence quality (read 2) of libraries prepared using ‘N’(0–10) spacer-linked V3-V4 primers without PhiX Spike-in

To evaluate the result obtained, we applied our approach to prepare 16S V3-V4 amplicon library from mock microbial community DNA standards (ZymoBIOMICS™) using standard illumina V3-V4 primers as well as our proposed ‘N’ (0–10) spacer-linked primer pool. 250 bp PE sequencing run was performed on illumina MiSeq platform using nano V2 kit producing Read 1 of 270 bp reads and Read 2 of 240 base pairs. The average quality scores (Q30) were 95.30% for libraries prepared with our proposed method without PhiX spike-in (Run 1, Fig-S3-B) and 94.10% using standard illumina V3-V4 primers with 20% PhiX spike-in (Run 2, Fig-S3-A). The quality metrics were further analysed using FastQC software (Ver: 0.11.8) for a better understanding and comparison (Fig-2).

**Fig. 3:**
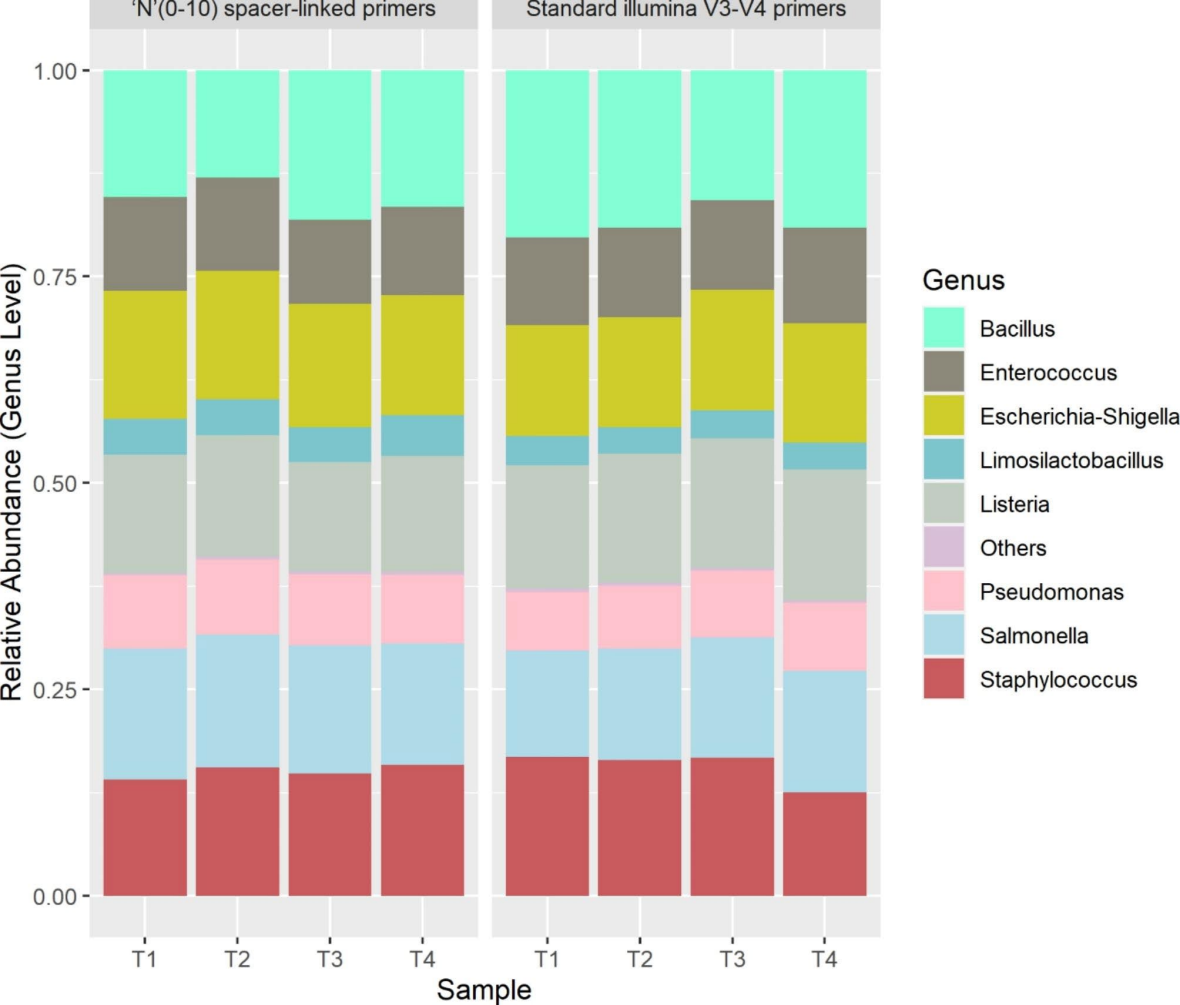
Comparison of relative abundance of bacterial community of mock sample (ZymoBIOMICS™ microbial community DNA standard) library prepared using Standard illumina V3-V4 primers versus ‘N’ (0–10) spacer-linked primers. T1, T2, T3 and T4 represent four technical replicates

Trimming of ‘N’ (0–10) spacers from the reads was performed using an in-house developed python based software MetReTrim (version 1.0, recommended) for the mock ‘N’ (0–10) spacer-linked library (check Methods section for algorithm, installation and usage details). The trimmed reads were found to be ≥ 97% of total reads. DADA2 pipeline (Ver: 1.24.0) was used for the analysis of generated data. While running the pipeline the truncation length parameter was manually selected based on the overall read quality profile. We couldn’t use Figaro, due to the variable length of our reads after heterogeneity spacers trimming. The optimized truncation parameters we used in our analysis hold true in most of the cases including low diversity samples. More than 95% of the reads were found to be non-chimeric in both the methods. Read counts were tracked throughout different crucial steps during the analysis to confirm no significant losses were seen in any of these steps (Table S1). Final Genus level abundance data was generated and compared in this study between the runs (Fig-3).

**Fig. 4:**
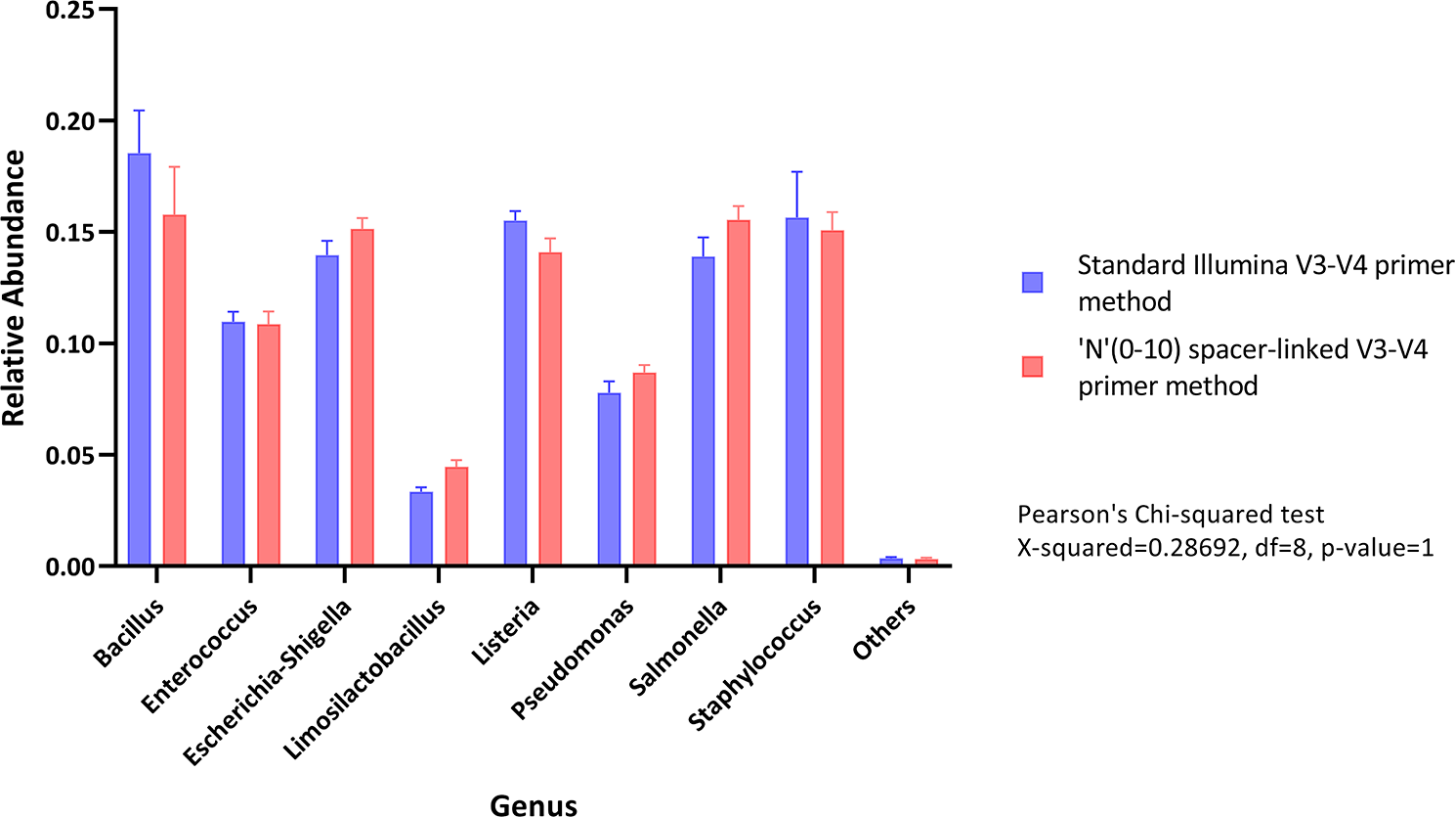
Pearson’s Chi squared test on means of relative abundance of technical replicates of Standard illumina V3-V4 primer method and ‘N’(0–10) spacer-linked V3-V4 primer method

**Fig. 5:**
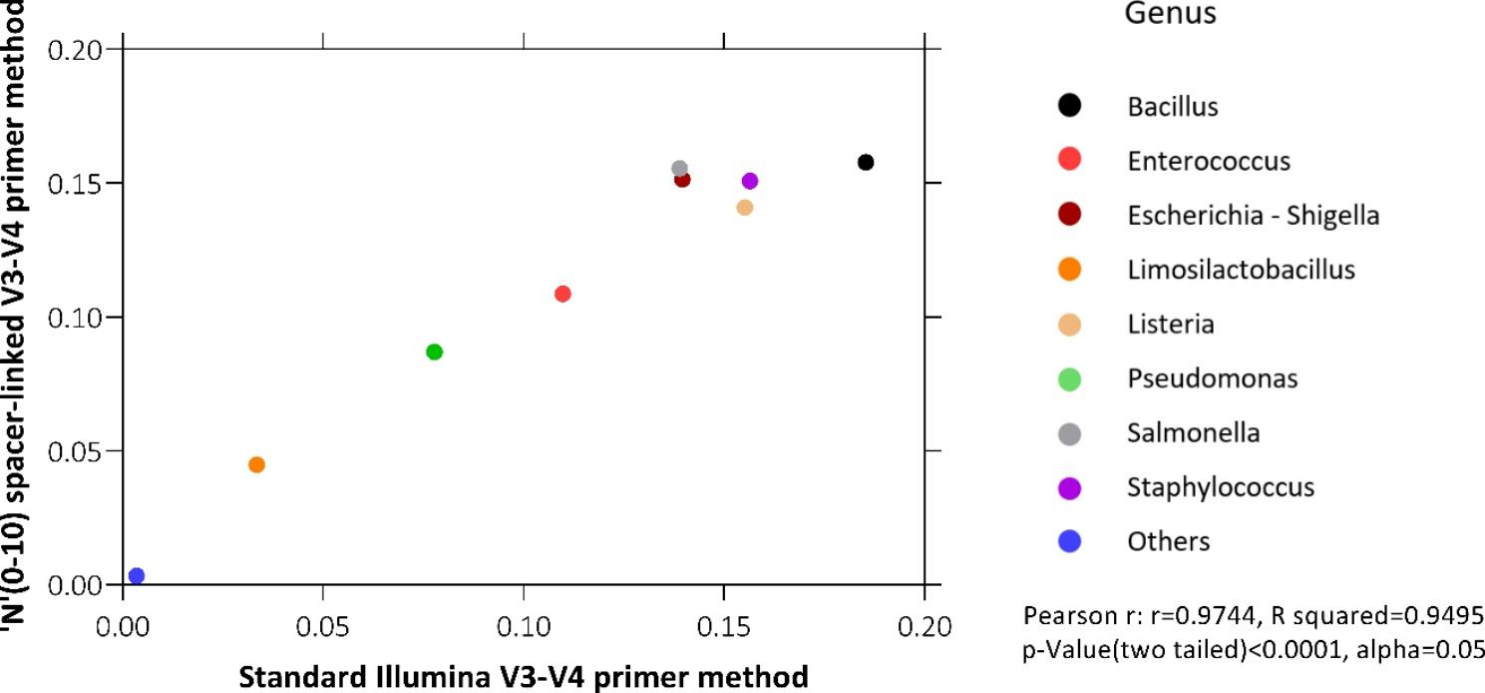
Two tailed t-test for correlation performed on Standard illumina V3-V4 primer method and ‘N’ (0–10) spacer-linked V3-V4 primer method

Through Pearson’s chi squared test, we found that there was no significant difference in the relative abundance between both the runs and all the technical replicates (Fig-4, X-squared = 0.28692, df = 8, p-value = 1). Further, we found out that genus composition of both the runs were highly similar (Fig-5, Pearson r = 0.9744, R-squared = 0.9495, p-value < 0.0001, alpha = 0.05).

Only difference we observed during our study compared to the ZymoBIOMICS™ handbook (https://www.bioscience.co.uk/userfiles/pdf/ZymoBIOMICS%E2%84%A2%20Microbial%20Community%20Standard%20.pdf) is that *Limosilactobacillus* genus was represented instead of *Lactobacillus* genus in both the methods. This is consistent with a recently proposed reclassification of genus *Lactobacillus* into 23 novel genus, including *Limosilactobacillus* [16].

We also performed variant calling analysis using Geneious Prime software (Version 4) taking the E.Coli 16S rRNA gene (GenBank: J01859.1) V3-V4 region as a reference. The analysis identified two SNP’s at the same loci with the same transition of bases in both the datasets i.e., mock dataset with our method and standard illumina method with PhiX spike-in. (Table-S2)

## Discussions

Amplicon sequencing leverages ultra-deep sequencing of PCR products for various kinds of application which includes variant identification and phylogenetic studies. Most commonly used amplicon sequencing application is for 16 S metagenomics where we target the variable regions interspersed between conserved regions of the 16S rRNA gene. In this study, we designed heterogeneity primers by adding ‘N’ (0–10)spacers to 5’end of 16S V3-V4 specific forward and reverse primers to generate amplicons with complex base diversity due to the frameshift effect. We verified that our method introduces base variability within individual libraries leading to effective laser registry without the need for PhiX spike-in. The overall improvement in read quality ensures reads of longer length retained after trimming. Pooling the primers for library preparation simplifies the experimental setup, and the design of modified primers becomes simpler and user friendly as they need to just introduce ‘N’ (0–10) spacers upstream of the primer sequences.

We compared our method with previously published methods that addressed diversity issues with single amplicon sequencing in illumina platforms (Table 2). Among some notable prior studies, Wu et al. [13] tried to shift the sequencing frame of amplicons by using spacers of 0–7 bases, however, in our studies we observed that spacers consisting of 7 bases are not sufficient to resolve the issue of unbalanced base distribution and requires a higher percentage of PhiX spike-in. Jensen et al. [9] designed heterogeneity primers by adding specific nucleotide bases to 16S V3-V4 primers to create 10 oligonucleotide sets and carried out MiSeq run with 10% PhiX. This approach requires the preparation of a minimum of 10 libraries to be pooled to generate base diversity. Adopting this strategy to other amplicon library preparation needs a careful designing of heterogeneity spacers to ensure base diversity by considering the sequence of primers used to amplify the gene of interest. Jensen et al. [9] also reported a significant drop in quality scores at 185–189 positions in sequence reads. In contrast, our method shows higher quality Q scores up to 265 bases in Read 1 and 235 bases in Read 2 and a slower drop thereafter (Fig-2 C and 2D). Holm et al. [3] performed 16S metagenomics runs with 5% PhiX spike-in using 2-step PCR library preparation with (0–7) heterogeneity spacer strategy and compared it between HiSeq (300 bp PE) and MiSeq (250 bp PE) platforms. Wherein, their quality of reads was better in Hiseq compared to MiSeq significantly. Moreover, the Quality score of the MiSeq reads started to drastically decline after 140 bases in read 1 and after 30 bases in read 2. In comparison, our study presented high-quality paired-end sequencing run on MiSeq without PhiX spike-in and also shows that a minimum of (0–10) base heterogeneity spacer is needed to resolve the issue of base diversity.

**Table 2:** Comparison with previously published methods that addressed diversity issues with single amplicon sequencing in illumina platforms

Approach	Spacer/Tag Design	Frame Shift	PCR strategy	Chimera potential	PhiX Spike-In	Mock Evaluation	Position in reads where Q score drop < 30	Run Metrics	Experimental cost	Reference
Random tags and spacer	Complex^a^	1 to 5	One step PCR (34)	More	No	No	Not mentioned	Cluster passing filter = 94%, 89.5% bases > Q30	High^c^	Lundberg et al. 2013 [1]
Improved dual-indexing approach with heterogeneity spacer	Complex^a^	1 to 7	One step (30)	More	~ 8–16% (Avg)	Yes	Not mentioned	~ 85–93% bases > Q30 (Avg)	High^c^	Fadrosh et al. 2014 [12]
Phasing amplicon sequencing (PAS)	Complex^a^	1 to 7	Two step (10, 20)	Less	10–20%	Yes	Read one – 250–251 bases Read two – 180 bases	93.3% bases > Q30	High^c^	Liyou Wu et al. 2015 [13]
Triple-index amplicon sequencing	Complex^a^	1 to 7	Two Step (25/30/35, 5/10)	Less	10%	Yes	Not Mentioned	Not mentioned	High^c^	Muinck et al. 2017 [7]
2-step PCR library preparation with heterogeneity spacer	Complex^b^	1 to 7	Two Step (20, 10)	Less	20%	Yes	Miseq Reads Read one – 148 bases Read two – 31 bases	Not mentioned	Moderate^d^	Holm et al. 2019 [3]
Target region amplification with a series of 10 primers	Complex^a^	1 to 7	Two step (25, 8)	Less	10%	No	Read one – 185 bases Read two-160 bases	Not Mentioned	High^c^	Jensen et al. 2019 [9]
Pool of ‘N’ (0–10) spacer-linked target specific primer	Simple^b^	1 to 10	Two step (25, 8)	Less	No	Yes	Read one – 265–270 bases Read two – 235–240 bases	Cluster passing filter = 97.5%, 95.3% bases > Q30	Low^e^	This manuscript

^a^Complex – Required careful planning to design of spacer/tag to maintain base balance at each position, increased workload in case of library preparation per sample, complex design of primer/tag/spacers/indices. (Wherever applicable)

^b^Simple – Simple design of primer/spacer/tag making the adaptability of the method easy to use even by non-experts

^c^High – Experimental setup tedious (requires 6–8 samples to be pooled for confirming base complexity) and PhiX spiking reduces the overall sequence read throughput and multiplexing options in a run

^d^Moderate – Experimental setup simple but requires PhiX spiking

^e^Low – Experimental setup simple, faster, base complexing within individual library and no need for PhiX spiking allows to multiplexing of more number of samples in a run

To corroborate our strategy for the amplification biases, if any introduced due to the modifications made in our primers, we compared the ZymoBIOMICS™ microbial community DNA standard between standard illumina V3-V4 primers method with 20% PhiX and our method without PhiX spike-in. While, because of the variations in loading concentration, we observed the changes in the yield between our method and standard illumina method. We did not find any significant variation in terms of relative abundance of genus between the two methods (Fig-3, 4 and 5). Also, we were able to successfully complete this study with 250 bp PE run instead of 300 bp PE run maintaining more than sufficient overlap during merged read formation in DADA2. Our method is also compatible and consistent with 300 bp PE run which may be required for specific experiments.

## Conclusion

The use of ‘N’ (0–10) spacer-linked primers generate nucleotide distinctiveness within individual libraries at each base resulting in better identification of clusters during library sequencing run and enhance confidence in nucleotide base calls. This method eliminates the need for PhiX spike-in for single amplicon sequencing on illumina MiSeq platform. This allows for sequencing of more number of samples in a run and a reduction in the overall cost. Given that Illumina sequencing works on SBS chemistry irrespective of the platform (such as HiSeq, MiSeq, NextSeq, NovaSeq, etc.) we propose that this strategy of using ‘N’ (0–10) spacer-linked primer design can be adopted for generating high-quality single locus amplicon sequencing in a high throughput manner across the illumina platforms subject to further validation.

## Methods

For complete and detailed methods followed in this study kindly refer to the supplementary information.

### Primer design

Bacterial 16S rRNA V3-V4 region was targeted to study the efficiency of ‘N’ spacer-linked primers. The primer contains illumina adapter overhang sequence (blue), ‘N’ (0–10) spacer region (red), and Target Gene-specific primer (green). (Fig-1; Tables 3 and 4). The primers were ordered as standard desalted PCR primers. Forward and Reverse primer stocks were diluted to 5µM and equal volumes of each forward and reverse primer were pooled together. For freshly ordered primers, it is recommended to check the efficiency of each primer before pooling them. Towards this we performed PCR using a control template and any combination of forward and reverse primer in a total 11 PCR reaction setup.

**Table 3:**
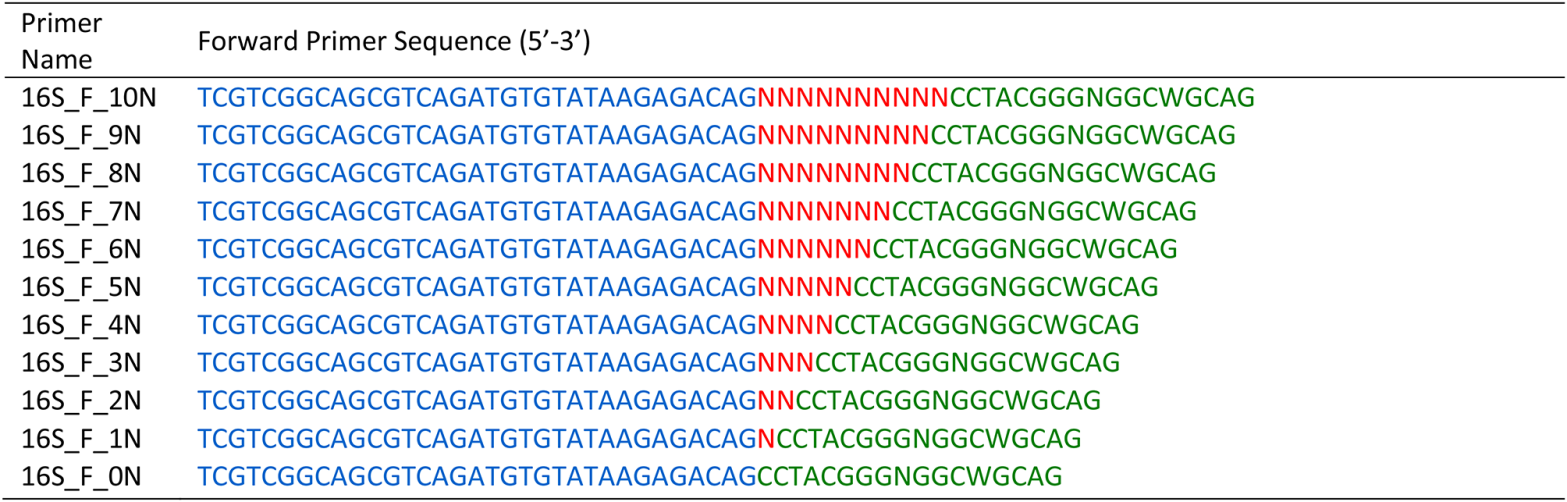
Forward ‘N’ spacer-linked primers required for the First round PCR, where ‘N’ represents random bases

**Table 4:**
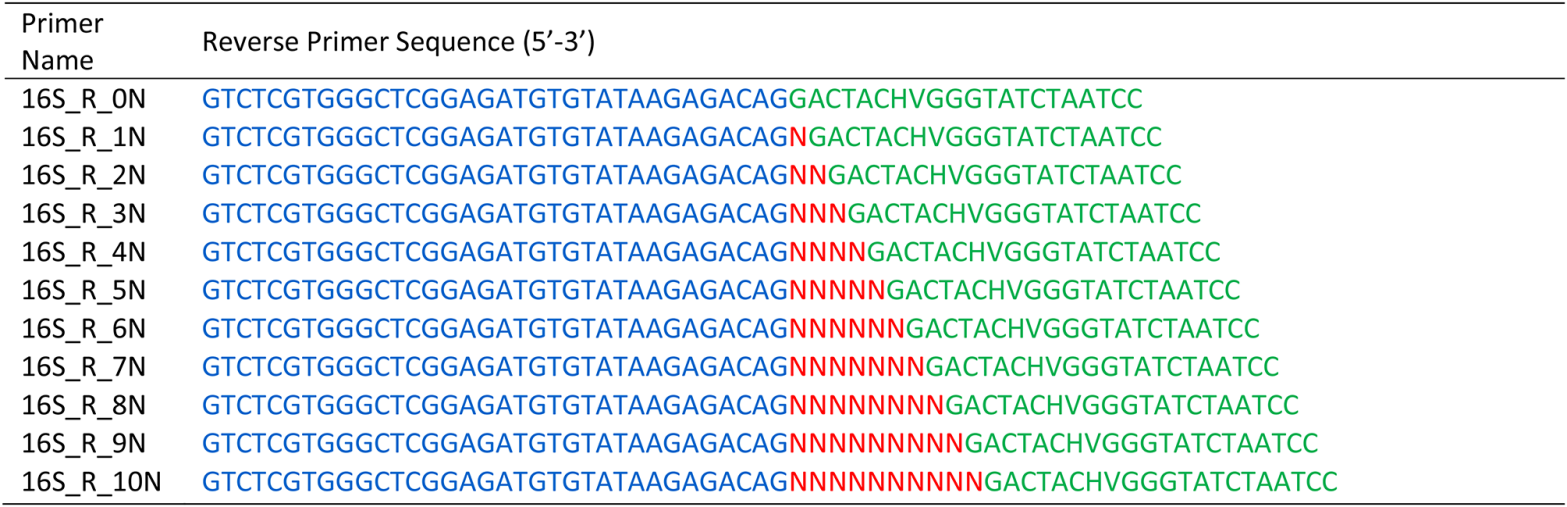
Reverse ‘N’ spacer-linked primers required for the First round PCR, where ‘N’ represents random bases

### Library preparation protocol

For both the methods (Standard illumina V3-V4 primer method and ‘N’ (0–10) spacer-linked V3-V4 primer method), four technical libraries were prepared from 2ng of mock community DNA (ZymoBIOMICS™ Microbial community DNA standard). The following reaction setup were used for first PCR amplification of 16S V3-V4 region: 4µL of mock community DNA, 1µL of forward primer or primer pool, 1µL of reverse primer or primer pool, 12.5 µL of KAPA HiFi HotStart Ready Mix (Cat. No.: KK2602, Roche), rest of the volume was made up to 25µL with Nuclease free water. The conditions used for the PCR reactions are as follows: Initial Denaturation at 95 °C for 3 min, followed by 25 cycles of 95 °C for 30s, 55 °C for 30s, 72 °C for 30s, final extension at 72 °C for 5 min, and then hold at 4 °C. The PCR amplicons clean-up was performed using Ampure XP Reagent (Cat. No: A63881, Beckman Coulter) with 0.8X concentration. The purified amplicons were then quantified using Qubit DNA HS reagent. The amplicon size was verified on High sensitivity D1000 screen tape (Agilent Technologies, Tapestation 4200). For the V3-V4 region expected size after PCR is ~ 540 bp and ~ 560 bp for standard illumina V3-V4 primer method and our method respectively (Fig-S4). With the purified first PCR product, a second PCR was setup. This step adds Index 1 (i7) and Index 2 (i5) sequences to generate uniquely tagged libraries by amplifying the target gene amplicons using illumina Nextera XT Index Kit V2 (Cat. No: FC-131-1002, Illumina). Illumina’s index adapters pooling guide was referred for the selection of compatible primer combinations. In TruSeq index plate fixture the Nextera XT V2 index 1 primer (i7) tubes were arranged horizontally in 1–12 fashion and Nextera XT index 2 primer (i5) tubes vertically in 1–8 fashion. 2µL of purified product was transferred to a new 96 well PCR plate and was placed on TruSeq index plate fixture, followed by addition of 5 µL Index primer 1, 5 µL Index primer 2 and 25 µL of KAPA HiFi HotStart Ready Mix. The following PCR program was carried out: initial denaturation at 95 °C for 3 min, followed by 8 cycles of 95 °C for 30s, 55 °C for 30s, 72 °C for 30s, final extension at 72 °C for 5 min, and then hold at 4 °C. The indexed amplicons clean-up was then performed with 1X Ampure XP reagent and the purified libraries were again quantified using the Qubit DNA HS reagent kit. The final library was then checked on Tapestation 4200 using high sensitivity D1000 screen tape to verify the size. The expected size for V3-V4 Region is ~ 610 bp and ~ 620 bp for standard illumina V3-V4 primer method and our method respectively (Fig-S4).

### Library normalization and pooling

The library concentration was calculated in nM, based on the average size and concentration of the library using the illumina pooling calculator (https://support.illumina.com/help/pooling-calculator/pooling-calculator.htm). The final library was then diluted using 10 mM Tris-HCl pH 8.0 to 2 nM. The concentration of the pooled library was then verified and the nM was calculated considering the average size of all libraries, which should be ~ 2nM.

### Library denaturing and MiSeq loading

The MiSeq Reagent Nano Kit v2 (500-cycles, Cat No: MS-103-1003) was thawed at room temperature as per the manufacturer’s guidelines. 0.1 N NaOH (pH 14.0) was freshly prepared from 2 N stock. 10 µL of 2nM pooled libraries was combined with 10 µL of 0.1 N NaOH and incubated at room temperature for 5 min to denature the libraries. 980 µL of pre-chilled HT1 buffer was then added to result in a 20pM of denatured library. It was then further diluted to 6.0 pM and 7.5 pM as the final loading concentration for our method and standard illumina method, respectively, and taken forward for subsequent sequencing. Each pool was run on MiSeq platform with 250 bp paired end reads and demultiplexed into individual Fastq datasets with MiSeq inbuilt algorithm.

### ’N’ (0–10) spacer trimming

In-house python software “MetReTrim” was written to trim the heterogeneity ‘N’ spacers from the 5’ end of the reads. The algorithm looks for the given unique primer sequence(s) in each read and allows up to N number of mismatches during the search (-m N option). Once the primer sequence is located, all the bases before the start of the primer sequence are trimmed. The primer sequence could be retained or dropped in the processed reads (-k option). Two files are generated in the output directory- (1) fastq file containing the trimmed reads and (2) fastq file containing untrimmed reads. The untrimmed reads are a result of primer sequences in the reads having more than N number of mismatches or insertions and deletions. The software could be run as a command-line using the following syntax for paired-end reads:

MetReTrim -i < path to fastq files folder> -o < desired path to trimmed output> -f < primer sequence for forward read> -r < primer sequence for reverse read>.

Please visit the following link for details on how to download the software and other usage related information: https://github.com/Mohak91/MetReTrim. The software can be installed and used either manually or using containerisation techniques; support is available for both Docker and Singularity. The image file can be found at https://hub.docker.com/r/mohaksharda/metretrim.

In addition to the above version (recommended) of the software, we also introduce a beta version MetReTrimV2, for strictly advanced users aiming for extra sensitivity while primer searching before trimming. This includes an expanded set of functionalities: 1. Plotting bar plots to show the percentage of reads trimmed and untrimmed. (-v plot option), 2. Produce a csv file with a table for each sample type and percentage of trimmed and untrimmed reads. 3. Smith-waterman dynamic programming algorithm to align primer with reads to account for mismatches and gaps (called the non-stringent mode which is default): - match score, mismatch score, gap open penalty, gap extend penalty for primer and read respectively. All these can be adjusted by the user. (For more information, use -h option), 4. minimum read length allowed for trimming, 5. maximum gaps allowed for trimming and 6. 5’ offset allowed. Further details can be found at the github link of the software.

### Mock community comparison analysis

The data from both independent runs were analysed using the DADA2 pipeline (Ver: 1.24.0, https://github.com/kvirbhadra/New_method_NGGF). While running the pipeline filterAndTrim function parameters were optimised. The truncation length parameter was manually selected based on the overall read quality profile. We observed that truncLen = c(260,230), matchIDs = TRUE and maxEE = c(2.5) was the optimal fit for our study. The primers were trimmed at this stage for all reads irrespective of the method of library preparation. DADA2 performed denoising, merging, and chimera removal on sequences with default parameters. The pipeline tracked the read count through each and every step and assigned reads to ASVs. These ASV’s were used for downstream taxonomic analysis by aligning tothe SILVA database (Silva Ver: 138.1 – updated Mar 10, 2021). Taxonomic analysis was done using the phyloseq R package (Ver. 1.40.0). The taxa and seqtab.nochim data frames were exported in CSV format. Final Genus level abundance was generated and compared in this study for both the runs.

### Statistical analysis

To determine the significance of differences among microbial communities we performed Pearson’s chi squared test on the mean of all technical replicates of the two runs. To identify correlation if any between the microbial compositions of two methods, we performed two tailed t-test (Pearson r with confidence at 95%). All the statistical analyses were performed using GraphPad Prism (Ver: 9.4.0 (673)).

## Electronic supplementary material

Below is the link to the electronic supplementary material.

**Additional file 1:** Detailed Protocol

**Additional file 2:** Table S1

**Additional file 3:** Table S2

**Additional file 4:** Fig-S1

**Additional file 5:** Fig-S2

**Additional file 6:** Fig-S3 A

**Additional file 7:** Fig-S3 B

**Additional file 8:** Fig-S4

## Data Availability

All sequence data have been uploaded to the Sequence Read Archive (SRA) under BioProject number PRJNA866667.

## Abbreviations

‘N’ (0–10): Random Nucleotide varying in length from 0 to 10
DNA: Deoxyribonucleic acid
RNA: Ribonucleic acid
rRNA: ribosomal RNA
dNTPs: deoxy nucleotide triphosphates
A, T, G, C: Adenine, Guanine, Cytosine and Thymine
PE: Paired-end
ASV: Amplicon sequence variants
PCR: Polymerase Chain Reaction
HS: High Sensitivity
µL: Micro liter
µM: Micromolar
nM: Nanomolar
pM: Picomolar
