# Supplementary material for: High-quality single amplicon sequencing method for illumina MiSeq platform using pool of ‘N’ (0–10) spacer-linked target specific primers without PhiX spike-in
Source: BMC Genomics. 2023 Mar 23;24:141. doi: 10.1186/s12864-023-09233-4 (PMC10037784; doi:10.1186/s12864-023-09233-4)
Supplement: Supplementary file 1 — Additional file 1: Detailed Protocol [file 12864_2023_9233_MOESM1_ESM.docx]

**Detailed Protocol**

1. **Reagents**

| **Product** | **Supplier** | **Catalog Number** |
| --- | --- | --- |
| Nuclease-free water | Ambion | AM993 |
| Ethanol | Merck | 64-17-5 |
| AMPure-XP Magnetic Beads | Beckman Coulter | A63881 |
| Ultrapure 1 M Tris-HCl pH 8.0 | Invitrogen | 15568-025 |
| Qubit ds DNA HS assay kit | Invitrogen | Q32854 |
| KAPA HiFi HotStart Ready Mix | Kapa Biosystems | KK2602 |
| Agilent DNA 1000 Kit | Agilent | 5067-5584 |
| Nextera XT Index kit V2 Set A | Illumina | FC-131-2001 |
| NaOH | Sigma | 1091361000 |
| MiSeq reagent cartridge and Flow cell | Illumina | MS-103-1003 |
| pH Test Strips (7.0-14.0) | Sigma | P-4411 |

2. **Equipment**

| **Product** | **Supplier** | **Catalog Number** |
| --- | --- | --- |
| Multichannel Pipette 0.5 - 10 µL | Eppendorf | 4922000021 |
| Multichannel Pipette 10 - 100 µL | Eppendorf | 4922000048 |
| Multichannel Pipette 30 - 300 µL | Eppendorf | 4922000064 |
| Plate centrifuge | Eppendorf | 5430R |
| PCR machine | Bio-Rad | 1851196 |
| Thermomixer | Eppendorf | 5382000015 |
| Sealing mat | Axygen | AM-96-PCR-RD |
| TruSeq Index Plate Fixture | Illumina | FC‐130‐1005 |
| Miseq Platform | Illumina | SY-410-1003 |
| Magnetic stand 96 | Invitrogen | AM10027 |
| 96 well plate | ThermoFisher | SP-0446 |
| Qubit fluorometer | Invitrogen | Q33238 |

3. **Primer design**

Bacterial 16S V3-V4 region was targeted to study the efficiency of ‘N’ spacer primer. The primer contains Illumina adapter overhang sequence (blue), ‘N’ spacer region (red), and Target Gene-specific primer (green). (Fig.1, Table 3 and Table 4). The primers were ordered as standard desalted PCR primers. Forward and Reverse primer stocks were diluted to 5μM and equal volumes of each forward and reverse primer were pooled together. For freshly ordered primers, it is recommended to check the efficiency of each primer before pooling them. Perform PCR using a control template and any combination of forward and reverse primer in a total 11 PCR reaction setup.

1. **Detailed Library Preparation Protocol**
   1. *First-round PCR-Amplifying target gene*

Assemble the following components per reaction

| **Reagents** | **Per well** |
| --- | --- |
| Microbial Genomic DNA (2 ng) | 4.0µL |
| Forward Primer Pool (5μM) | 1.0µL |
| Reverse Primer Pool (5μM) | 1.0µL |
| KAPA HiFi HotStart Ready Mix | 12.5µL |
| Nuclease Free Water | up to 25µL |

Use 96 well plate for more samples. Carry out the following PCR program:

1. 95°C for 3 min,
2. 95°C for 30s, 55°C for 30s, 72°C for 30s for 25 cycles
3. 72°C for 5 min, Hold at 4°C
   1. *Amplicon Cleanup:*
      1. Centrifuge the Amplicon PCR plate at 280 × g at 20°C for 1 minute.
      2. Vortex the AMPure XP beads for 30 seconds to make sure that the beads are evenly distributed
      3. Add 0.8X (20 µL) of AMPure XP beads to each well using a multichannel pipette, mix the beads by gently pipetting the entire volume 10-15 times.
      4. Incubate the plate for 5 minutes at RT
      5. Keep the plate on Magnetic stand for 5 minutes or until the supernatant is clear.
      6. Without disturbing the plate kept on Magnetic stand, discard the supernatant using Multichannel Pipette.
      7. With the Amplicon PCR plate on the magnetic stand, wash the beads by adding 150µL of freshly prepared 80% ethanol to each sample well. Incubate for 30 seconds. Carefully remove and discard the supernatant.
      8. Repeat the above step for a total of two washes. Using a multichannel pipette carefully remove the leftover ethanol from each well.
      9. With the Amplicon PCR plate on the magnetic stand, air-dry the beads for 2 mins. Do not over-dry the beads.
      10. Remove the Amplicon PCR plate from a magnetic stand, using a multichannel pipette resuspend the beads in 25 µL of 10 mM Tris-HCl pH 8.0 in each well.
      11. Mix the beads by gently pipetting the entire volume 10-15 times to ensure proper resuspension of beads.
      12. Incubate for 2 minutes at RT
      13. Place the plate on the magnetic stand for 5 minutes or until the supernatant is clear.
      14. Using a multichannel pipette, carefully transfer 22.5 µL of the supernatant to a new 96 well PCR plate.
      15. Quantify the amplicons using the Qubit DNA HS reagent kit.

[Optional] Verify the amplicon size on Tapestation using HS DNA 1000 Screen tape. For the V3-V4 region expected size after PCR is ~540-560 bp.

- 1. *Second-round PCR- Nextera XT Index Barcoding*

This step adds Index 1 (i7) and Index 2 (i5) sequences to generate uniquely tagged libraries by amplifying the target gene amplicons using Illumina Nextera XT Index Kit V2. Refer to Illumina’s Index Adapters Pooling Guide for the selection of compatible primer combinations. In TruSeq Index Plate Fixture arrange the Nextera XT Index 1 primer (i7) tubes horizontally from 1-12 fashion and Nextera XT Index 2 primer (i5) tubes vertically in 1-8 fashion. Transfer 2ul of purified product to a new 96 well PCR plate and place it on TruSeq Index Plate Fixture. Set up the following reaction using Multichannel pipette for adding Index primer 1 and primer 2:

| **Reagents** | **Per well** |
| --- | --- |
| Purified Amplicon | 2.0µL |
| Nextera XT Index Primer 1 | 5.0µL |
| Nextera XT Index Primer 2 | 5.0µL |
| KAPA HiFi HotStart Ready Mix | 25.0µL |
| Nuclease Free Water | up to 50µL |

Gently pipette up and down 10-15 times and seal the plate with septa. Carry out the following PCR program:

1. 95°C for 3 min,
2. 8 cycles of 95°C for 30s, 55°C for 30s, 72°C for 30s
3. 72°C for 5 min, Hold at 4°C
   1. *Index Amplicon Cleanup:*
      1. Centrifuge the indexed amplicon PCR plate at 280 × g at 20°C for 1 minute.
      2. Vortex the AMPure XP beads for 30 seconds to make sure that the beads are evenly distributed.
      3. Add 1X (50 µL) of AMPure XP beads to each well using a multichannel pipette, mix the beads by gently pipetting the entire volume 10-15 times.
      4. Incubate the plate for 5 minutes at RT.
      5. Keep the plate on Magnetic stand for 5 minutes or until the supernatant is clear.
      6. Without disturbing the plate kept on Magnetic stand, discard the supernatant using Multichannel Pipette.
      7. With the Amplicon PCR plate on the magnetic stand, wash the beads by adding 150µL of freshly prepared 80% ethanol to each sample well. Incubate for 30 seconds. Carefully remove and discard the supernatant.
      8. Repeat the above step for a total of two washes. Using a multichannel pipette carefully remove the leftover ethanol from each well.
      9. With the Amplicon PCR plate on the magnetic stand, air-dry the beads for 2 mins. Do not over-dry the beads.
      10. Remove the indexed Amplicon PCR plate from a magnetic stand, using a multichannel pipette resuspend the beads in 37.5 µL of 10 mM Tris-HCl pH 8.0 in each well.
      11. Mix the beads by gently pipetting the entire volume 10-15 times to ensure proper resuspension of beads.
      12. Incubate for 2 minutes at RT.
      13. Place the plate on the magnetic stand for 5 minutes or until the supernatant is clear.
      14. Using a multichannel pipette, carefully transfer 35 µL of the supernatant to a new 96 well PCR plate.
      15. Quantify the libraries using the Qubit DNA HS reagent kit.
      16. Check the final library on a Tapestation using HS DNA 1000 screen tape to verify the size. The expected size for V3-V4 Region is ~610-620bp.

4.5 *Library Normalization and Pooling*

Calculate library concentration in nM, based on the average size and concentration of the library using the illumina pooling calculator (<https://support.illumina.com/help/pooling-calculator/pooling-calculator.htm>) Dilute the final library using 10 mM Tris-HCl pH 8.0 to 4 nM. Aliquot 5 μL from each diluted library and mix aliquots for pooling libraries with unique indices. Depending on coverage needs, 96 or more than 96 libraries can be pooled for one MiSeq run. Check the concentration of the pooled library and calculate the nM considering the average size of all libraries. It should be ~2nM.

1. **Library Denaturing and MiSeq Loading**
   1. Keep the Miseq Reagent for thawing at RT.
   2. Freshly prepare 0.1N NaOH.
   3. Keep the thawed HT1 buffer in Ice.
   4. Combine the 10 μL of 2nM pooled library and 10 μL 0.1 N NaOH in a 1.5 ml tube to denature the library.
   5. Mix with pipette and vortex briefly.
   6. Centrifuge the sample solution at 280 × g at 20°C for 1 minute.
   7. Incubate for 5 minutes at room temperature to denature the library into single strands.
   8. Add 980 μL Prechilled HT1 buffer. Adding HT1 buffer results in a 20 pM denatured library.
   9. Dilute the 20 pM denatured library to the desired loading concentration based on Miseq kit.
   10. Load the denatured Library in Miseq reagent cartridge and start the sequencing run.
