# Supplementary material for: High-quality single amplicon sequencing method for illumina MiSeq platform using pool of ‘N’ (0–10) spacer-linked target specific primers without PhiX spike-in
Source: BMC Genomics. 2023 Mar 23;24:141. doi: 10.1186/s12864-023-09233-4 (PMC10037784; doi:10.1186/s12864-023-09233-4)
Supplement: Supplementary file 2 — Additional file 2: Table S1 [file 12864_2023_9233_MOESM2_ESM.docx]

**Table S1:** Read track data through DADA2 pipeline for both methods. Analysis was performed together as well as separately for both runs.

| **Method** | **Sample (in replicates)** | **input** | **filtered** | **denoisedF** | **denoisedR** | **merged** | **nonchim** |
| --- | --- | --- | --- | --- | --- | --- | --- |
| **Standard illumina V3-V4 primer method** | **T1** | 173594 | 151211 | 150831 | 150759 | 140089 | 132451 |
|  | **T2** | 216964 | 187940 | 187563 | 187355 | 175569 | 166531 |
|  | **T3** | 155156 | 135164 | 134770 | 134791 | 125178 | 118608 |
|  | **T4** | 197169 | 171701 | 171272 | 171167 | 159663 | 150791 |
| **‘N’(0-10) spacer-linked primer method** | **T1** | 150187 | 129559 | 128691 | 129206 | 123169 | 119347 |
|  | **T2** | 151358 | 131226 | 130905 | 130823 | 124652 | 119218 |
|  | **T3** | 87393 | 75139 | 74928 | 74830 | 71357 | 68950 |
|  | **T4** | 165410 | 141973 | 141283 | 141570 | 135668 | 131120 |
