# Supplementary material for: High-quality single amplicon sequencing method for illumina MiSeq platform using pool of ‘N’ (0–10) spacer-linked target specific primers without PhiX spike-in
Source: BMC Genomics. 2023 Mar 23;24:141. doi: 10.1186/s12864-023-09233-4 (PMC10037784; doi:10.1186/s12864-023-09233-4)
Supplement: Supplementary file 3 — Additional file 3: Table S2 [file 12864_2023_9233_MOESM3_ESM.docx]

**Table S2:** Comparison of SNP detected from Standard illumina V3-V4 primer method versus ‘N’ (0-10) spacer-linked primer method

| Method | Polymorphism type | *E. coli* 16S rRNA gene nucleotide position | Change | Average Quality | Variant Frequency | Variant P-Value |
| --- | --- | --- | --- | --- | --- | --- |
| Standard illumina V3-V4 primer method with 20% Phix Spike-in | SNP (transition) | 593 | T -> C | 34 | 31.1% | 0.00 |
|  | SNP (transition) | 599 | A -> G | 33 | 31.50% | 0.00 |
| ‘N’(0-10) spacer-linked primer method without Phix Spike-in | SNP (transition) | 593 | T -> C | 35 | 32.80% | 0.00 |
|  | SNP (transition) | 599 | A -> G | 34 | 32.90% | 0.00 |
