# Supplementary figures and images for: High-quality single amplicon sequencing method for illumina MiSeq platform using pool of ‘N’ (0–10) spacer-linked target specific primers without PhiX spike-in
Source: BMC Genomics. 2023 Mar 23;24:141. doi: 10.1186/s12864-023-09233-4 (PMC10037784; doi:10.1186/s12864-023-09233-4)

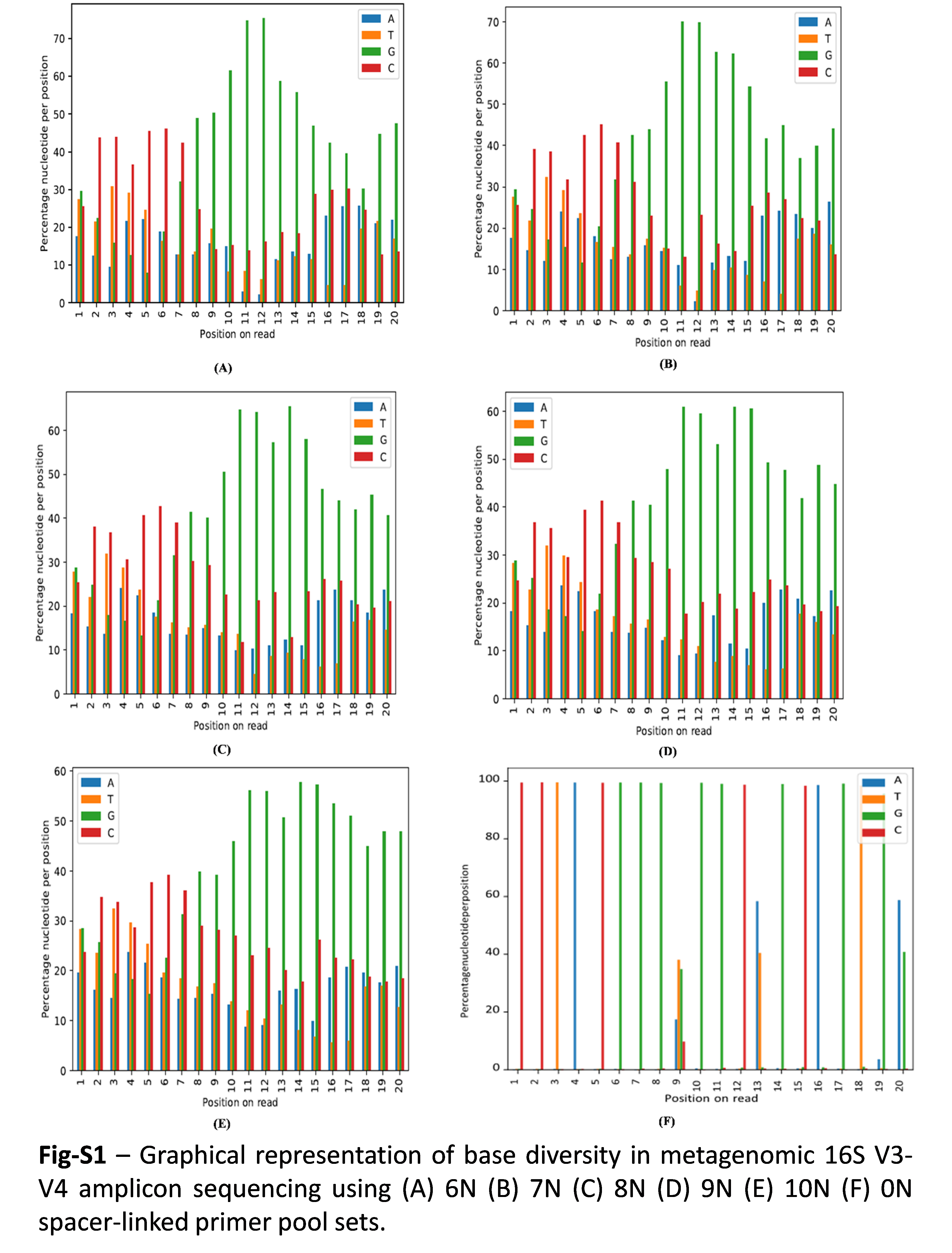

Supplement: Supplementary file 4 — Additional file 4: Fig-S1 [file 12864_2023_9233_MOESM4_ESM.png]

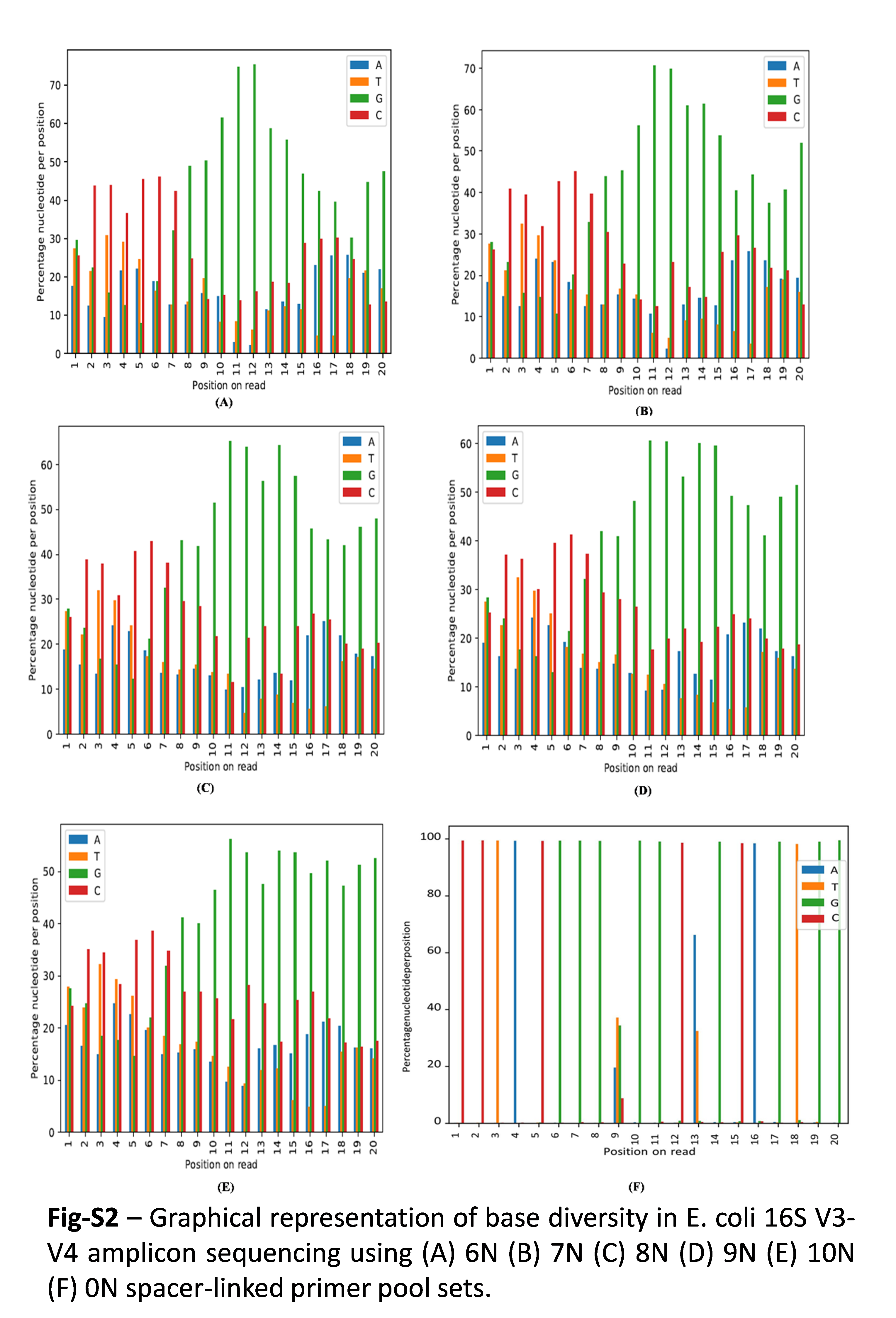

Supplement: Supplementary file 5 — Additional file 5: Fig-S2 [file 12864_2023_9233_MOESM5_ESM.png]

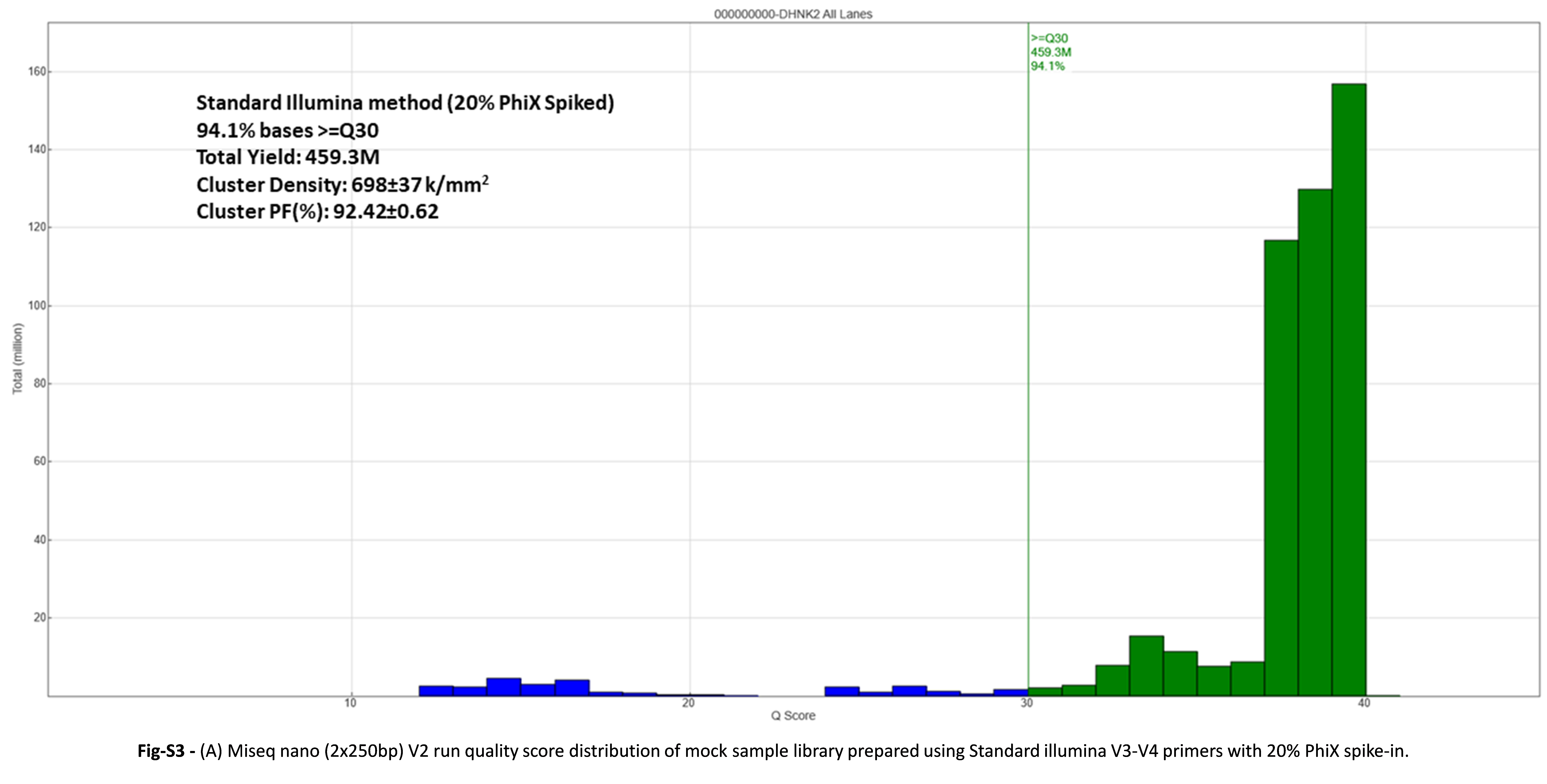

Supplement: Supplementary file 6 — Additional file 6: Fig-S3 A [file 12864_2023_9233_MOESM6_ESM.png]

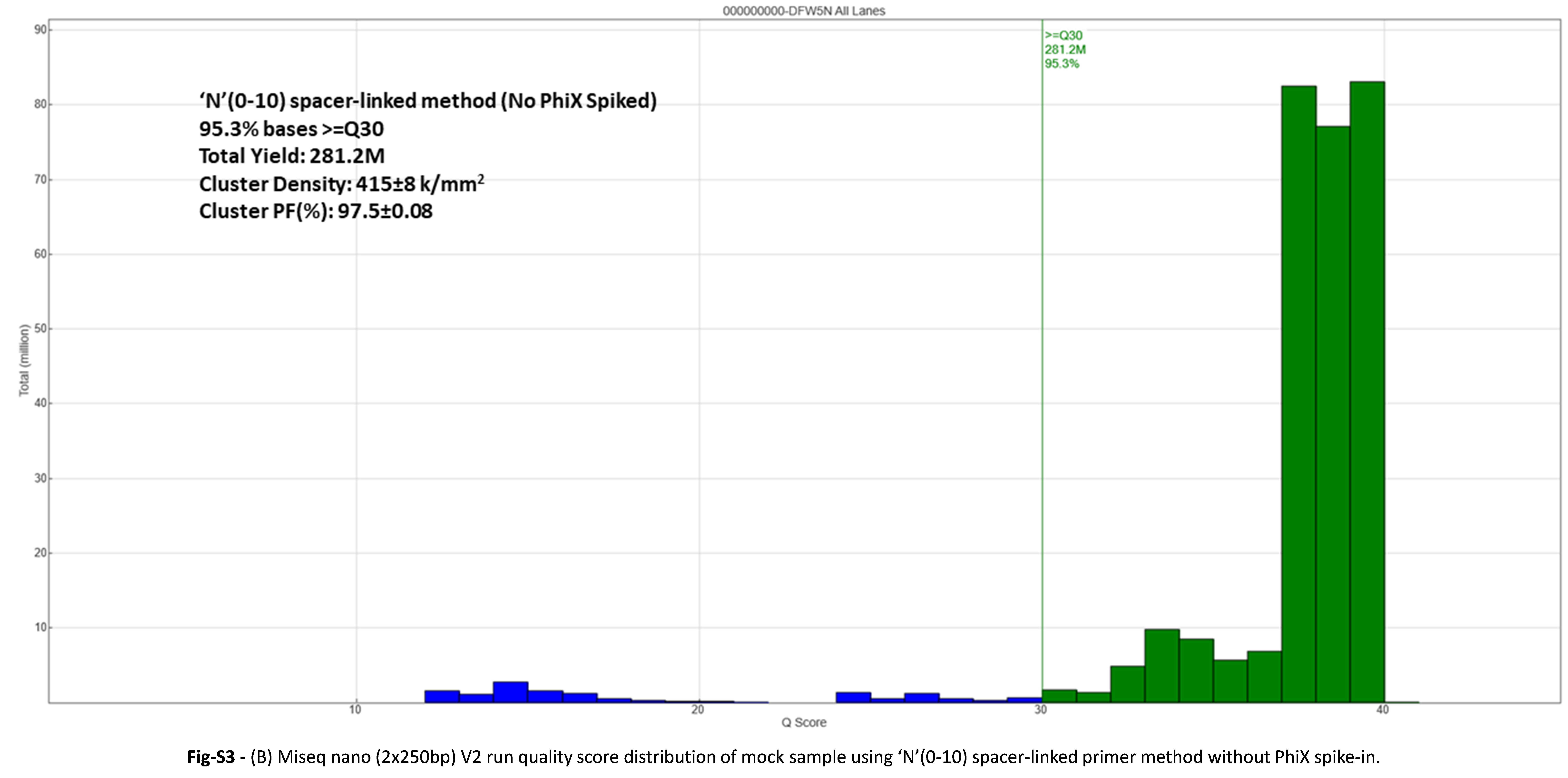

Supplement: Supplementary file 7 — Additional file 7: Fig-S3 B [file 12864_2023_9233_MOESM7_ESM.png]

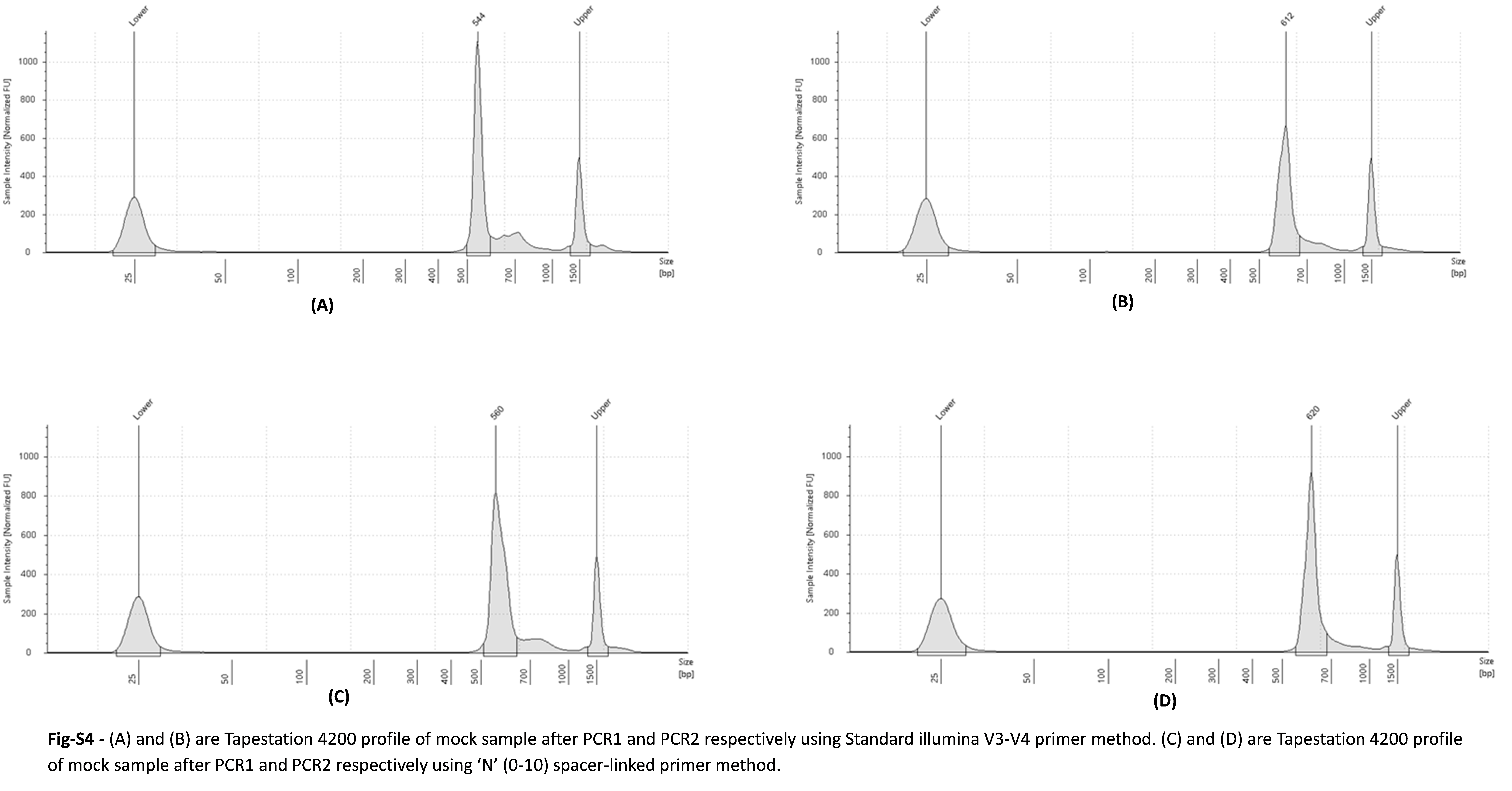

Supplement: Supplementary file 8 — Additional file 8: Fig-S4 [file 12864_2023_9233_MOESM8_ESM.png]
